# Supplementary material for: Marcus inverted region of charge transfer from low-dimensional semiconductor materials
Source: Nat Commun. 2021 Nov 3;12:6333. doi: 10.1038/s41467-021-26705-x (PMC8566515; doi:10.1038/s41467-021-26705-x)
Supplement: Supplementary file 1 — Supplementary Information [file 41467_2021_26705_MOESM1_ESM.pdf]

Supplementary Information

Marcus Inverted Region of Charge Transfer from Low-Dimensional Semiconductor Materials

Wang et al.

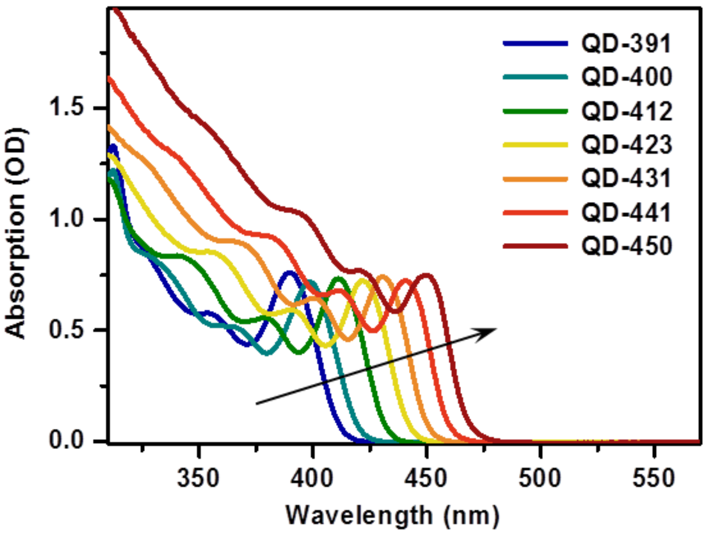

Supplementary Figure 1. UV-vis absorption spectra of CdS QDs with various sizes. They are labeled according to the positions of their first excitonic peaks.

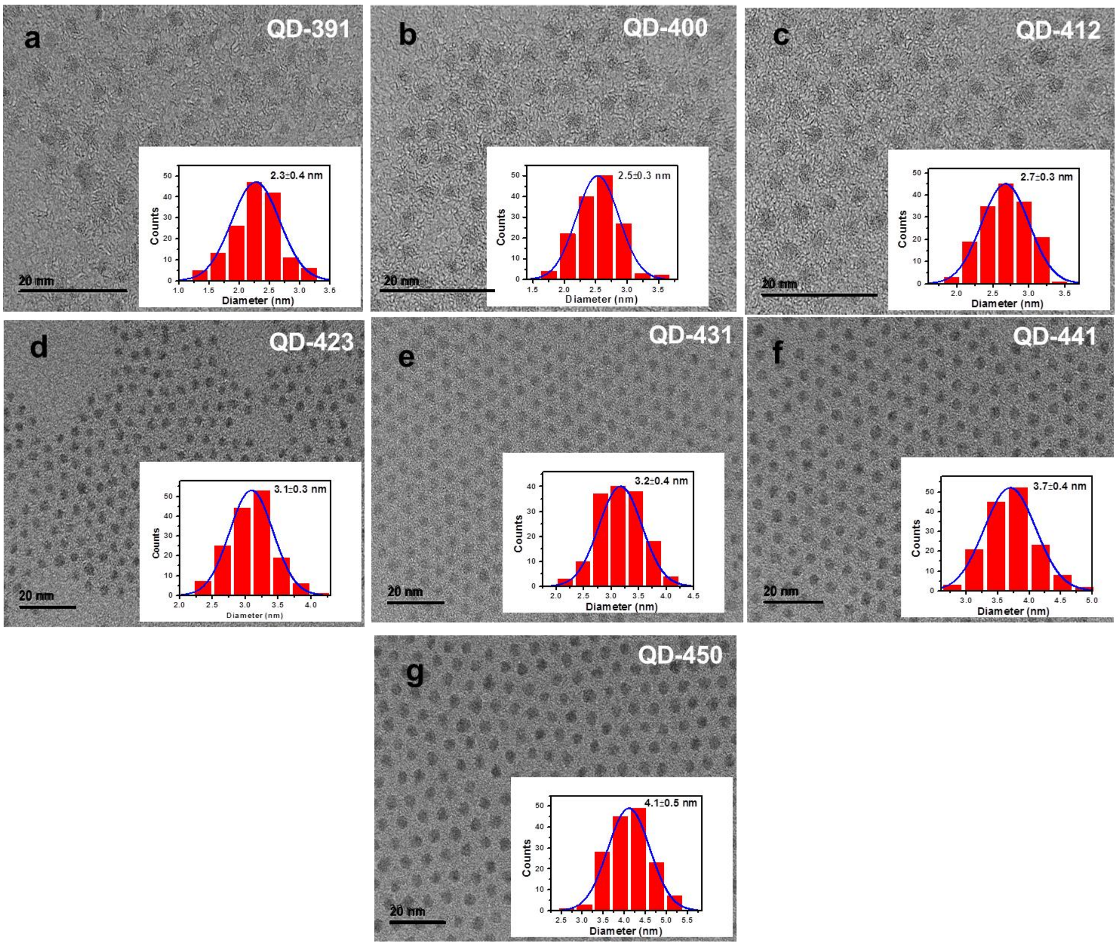

Supplementary Figure 2. TEM images of representative CdS QD samples. Insets are the statistical histograms of the diameters of QDs.

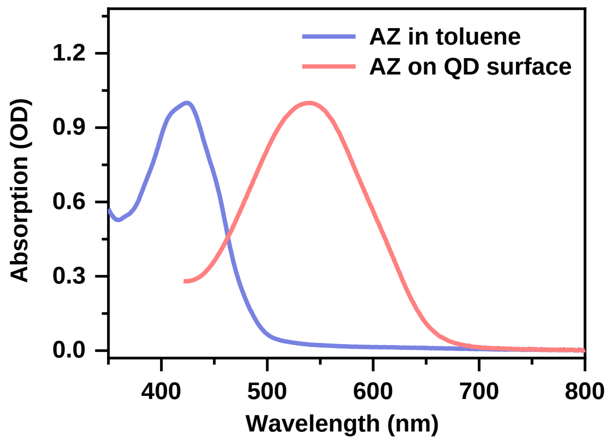

Supplementary Figure 3. Absorption spectra of free AZs in toluene and AZs adsorbed on CdS QD surfaces in hexane. The absorption spectrum of the latter was obtained by subtracting QDs from QD-AZ complexes.

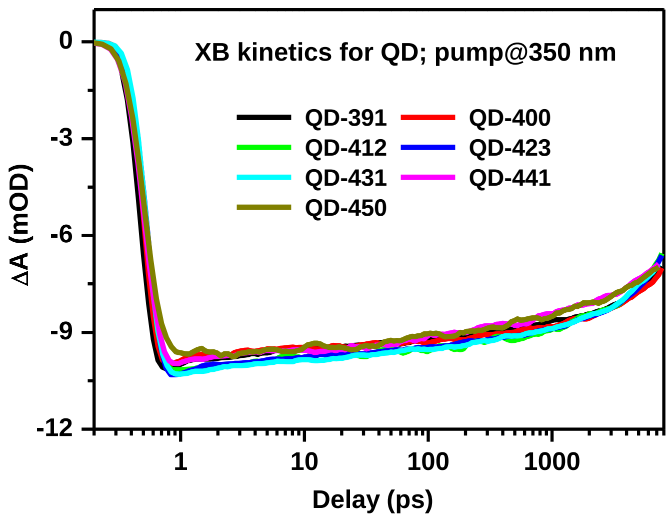

Supplementary Figure 4. XB kinetics for CdS QDs of various sizes excited at 350 nm. All samples show less than 30% decay within 8 ns, indicative of negligible electron trapping in this time window.

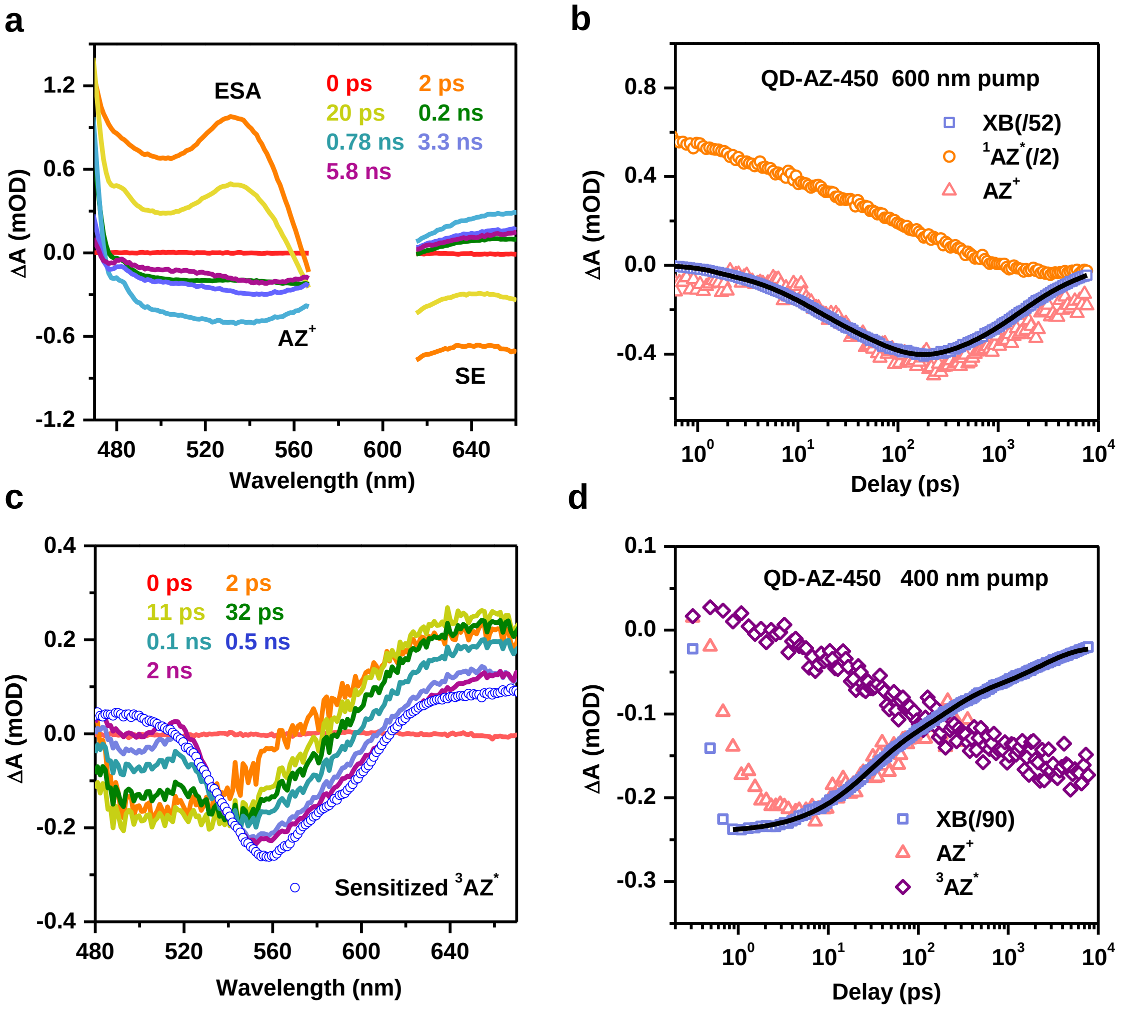

Supplementary Figure 5. TA of CdS QD-AZ-450 complexes pumped at 600 and 400 nm. (a, c) Time slices of the TA spectra for the enlarged view of AZ signals following (a) 600 nm and (c) 400 nm excitation respectively, featured by the excited state absorption (ESA), cation absorption (AZ^+), stimulated emission (SE) and triplet state absorption ($^3AZ^*$) of AZs. (b) TA kinetics of XB (blue squares, divided by factor of 52), $^1AZ^*$ (orange circles, divided by 2; monitored at 474 nm) and AZ^+ (red triangles; monitored at 565 nm) for QD-AZ complexes under 600 nm excitation. The decay of $^1AZ^*$ is accompanied by the growth of AZ^+ and QD XB, indicative of electron transfer from $^1AZ^*$ to QDs, and the ensuing decay of AZ^+ and QD XB is due to charge recombination (CR1) regenerating the ground-state QD-AZ. (d) TA kinetics of XB (blue squares, divided by 90), AZ^+ (red triangles; monitored at 531 nm) and $^3AZ^*$ (purple diamonds; monitored at 581 nm) for QD-AZ complexes under 400 nm excitation. The black solid lines are multi-exponential fits to XB kinetics. The ultrafast formation (in 2 ps) of AZ^+ indicates rapid hole transfer from excited QDs to AZs, and the following decay of AZ^+ and QD XB is accompanied with the formation of a long-lived $^3AZ^*$ feature (CR2).

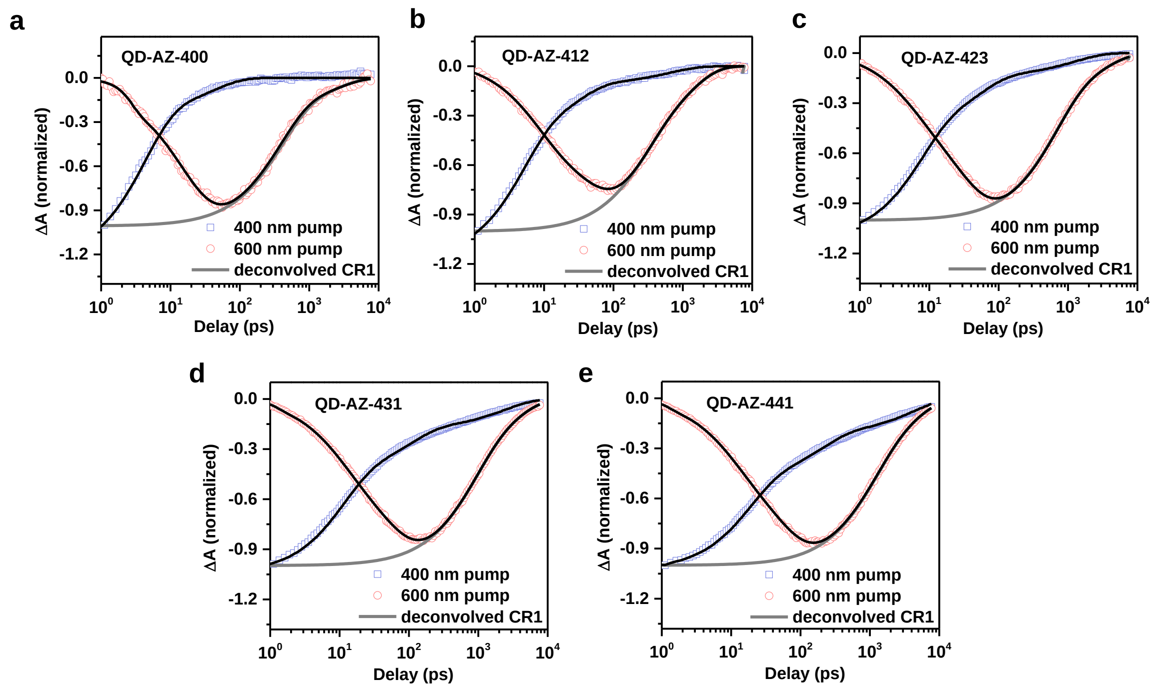

Supplementary Figure 6. The XB kinetics for CdS QD-AZ complexes with different QD sizes under both 400 and 600 nm excitations. The black solid lines are multi-exponential fits to XB kinetics. The kinetics of CR1 (gray lines) are obtained by deconvoluting the ET and CR1 kinetics.

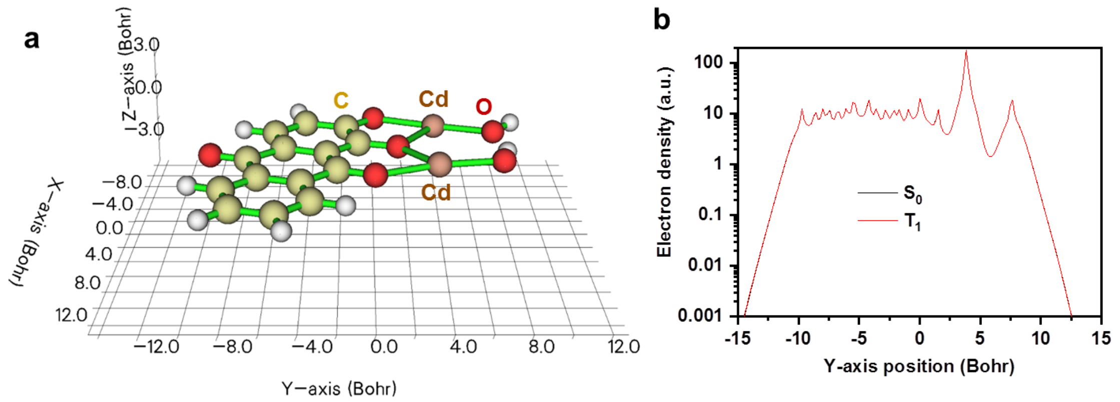

Supplementary Figure 7. DFT calculations. (a) The optimized geometry of the model compound of CdS-surface-bound AZ. The color codings of atoms are indicated except for H atom (white). Note the two hydroxyl groups attached to Cd are added for the purpose of balancing the charge. (b) Computed electron density distributions along the Y-axis (the normal of the QD-AZ interface) for S_0 (black) and T_1 (red) states by integrating along the other two axes.

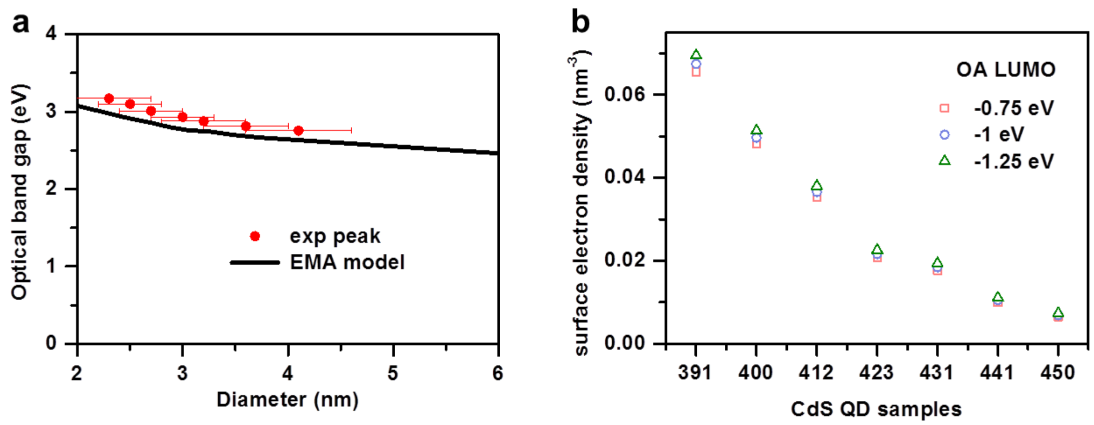

Supplementary Figure 8. EMA calculations. (a) Comparison of the optical gaps of CdS QDs calculated from the first quantized electron and hole levels and accounting for the electron-hole binding as a perturbation (black solid line) with the experimental data (red circles). The error bars represent the size distributions of the QDs. (b) Calculated surface electron densities ($|\Psi_s|^2$) with the OA LUMO at -0.75 eV (red squares). -1 eV (blue circles) and -1.25 eV (green triangles) *vs.* vacuum.

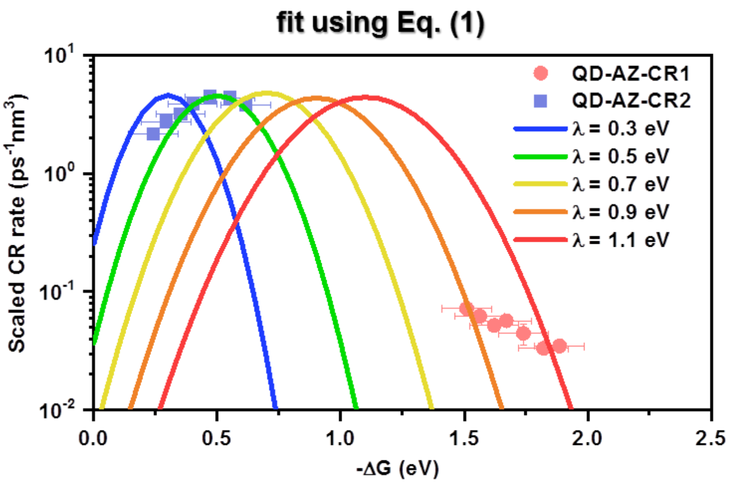

Supplementary Figure 9. Fitting the experimental CR-1 (red circles) and CR-2 rates (blue squares) as a function of size-dependent driving forces (-Δ*G*) in QD-AZ complexes. The colored solid lines are fits to Marcus theory in Eq. 1 with the total reorganization energy λ as the adjustable parameter. Horizontal error bars stand for the accuracy of the electrochemical experiments for energy levels (±0.1 eV), whereas vertical errors are the fitting errors of the CR rates.

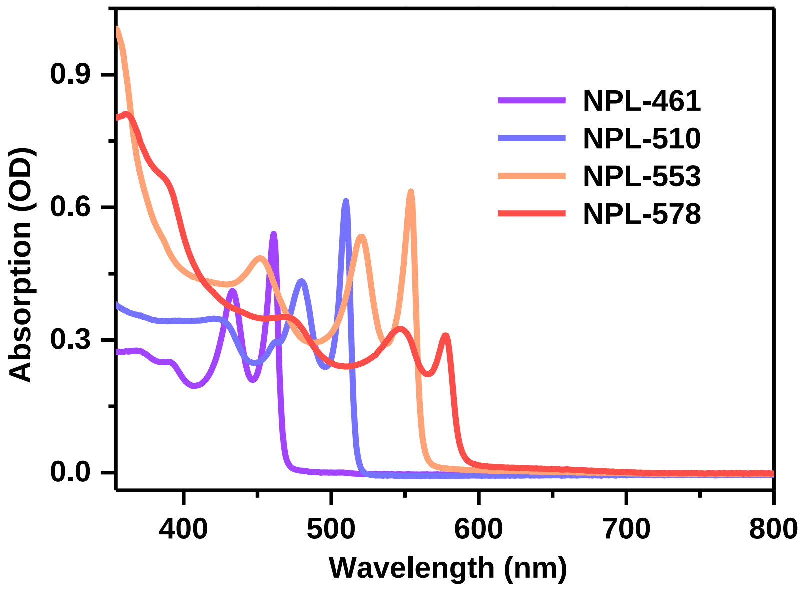

Supplementary Figure 10. UV-vis absorption spectra of CdSe NPLs with various thicknesses.

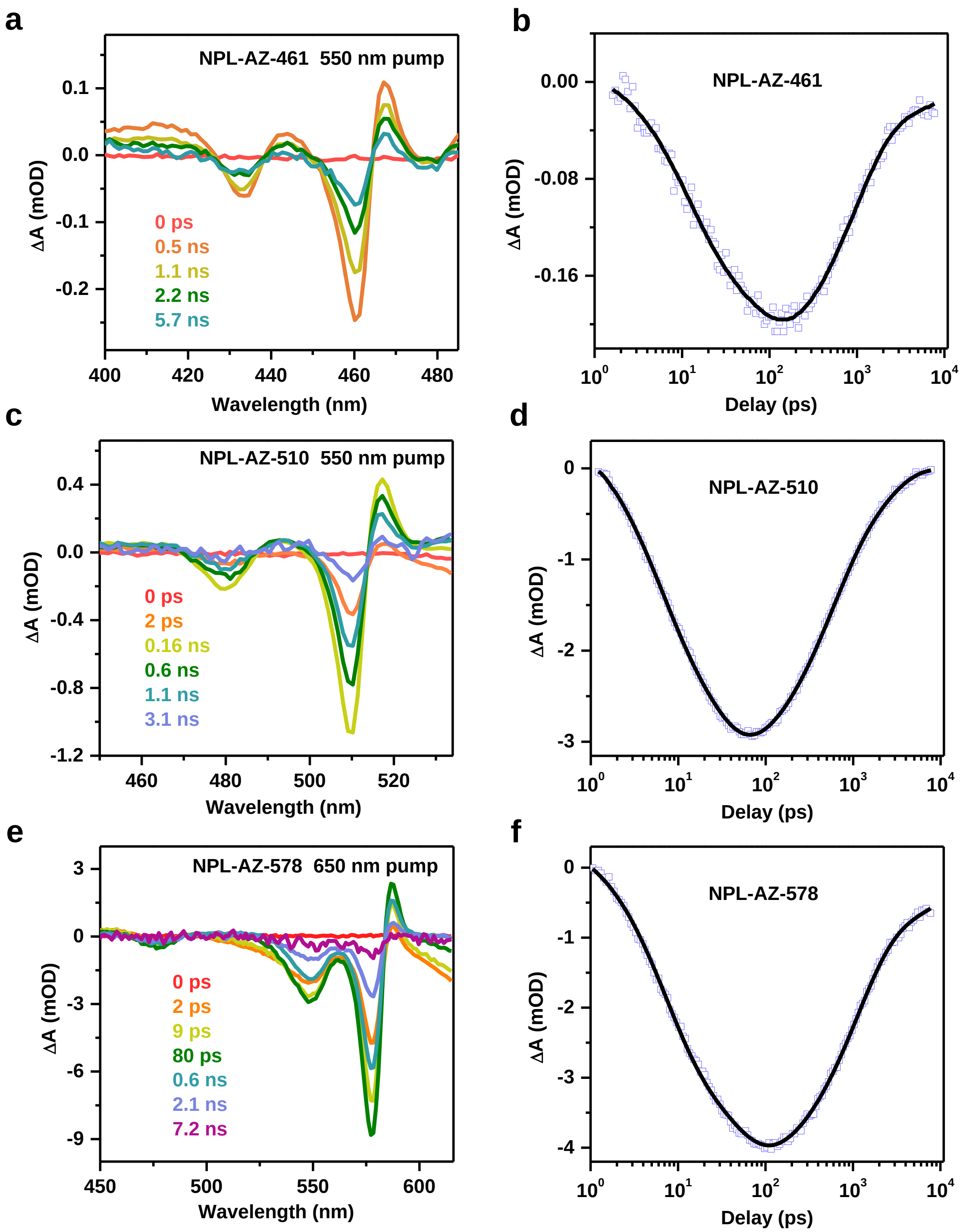

Supplementary Figure 11. TA spectra and kinetics for NPL-AZ complexes. (a, c, e) TA spectra measured for NPL-AZ complexes of different thicknesses excited at AZs. (b, d, f) ET and CR-1 kinetics for these samples (blue squares) and their multi-exponential fits (black solid lines).

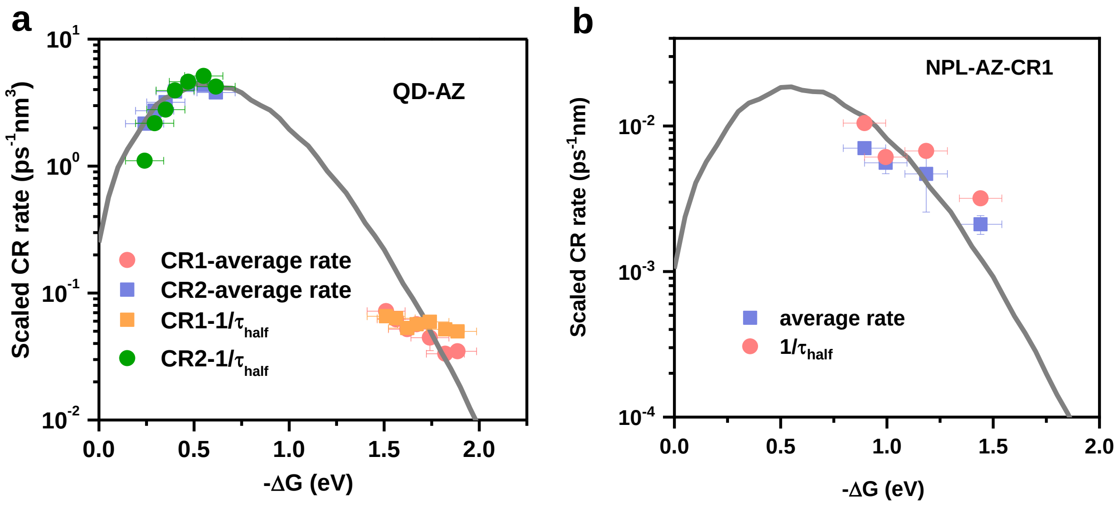

Supplementary Figure 12. Fitting using half-lifetimes. The plots of (a) CR-1 (orange squares) and CR-2 rates (green circles) for CdS QD-AZ complexes, and (b) CR-1 rates for CdSe NPL-AZ complexes (red circles) as a function of -Δ*G*, where CR-1 and CR-2 rates are obtained by the apparent half-lifetimes directly read from the kinetic curves (i.e., $1/\tau_{half}$ as the rate). The average rates used in the main text are also included. Both rates from half-lifetimes and average rates give consistent results that can be fitted to Marcus theory. Horizontal error bars stand for the accuracy of the electrochemical experiments for energy levels (±0.1 eV), whereas vertical errors are the fitting errors of the CR rates.

Supplementary Note 1. Spin-controlled charge recombination pathways in CdS QD-AZ complexes.

Detailed description and explanation of this observation are provided in our previous report.[1] This observation is established on the basis of the different spin-flip lifetimes of the electron ad hole in II-VI group QDs. Previous studies on spin relaxation in II-VI group QDs revealed that the hole spin lifetime was very short (sub-ps),[2,3] whereas the electron spin lifetime could be as long as ns in the absence of the hole.[4-6] This observation can be rationalized by that the Bloch function of the VB edge hole has an angular momentum of 1 (made from *p*-type atomic orbitals), enabling fast hole spin-flip via spin-orbit coupling.[7,8] On the other hand, both the Bloch (*s*-type atomic orbitals) and envelop ($1S_e$) parts of the CB edge electron wavefunctions have angular momenta of zero, inhibiting fast spin-flip via spin-orbital coupling;[9] spin-flip via phonon mediated scattering is also strongly inhibited, because of the sparsely-spaced electron levels at the CB edge arising from strong quantum confinement of electrons.[10]

When AZs are selectively excited (forming $^1AZ^*$), the charge separated state should mostly maintain a spin-singlet configuration, i.e., $[QD^- \text{-} AZ^+]^1$. If the spin-flip rate of the electron in the CB edge of QDs is slower than the rate of CR1, which is satisfied in the current CdS QD-AZ system, the major recombination product is the ground state QD-AZ complexes. When QDs are selectively excited, the photogenerated exciton undergoes rapid (sub-ps) hole spin-flip, generating an excitonic state dominated by a spin-triplet-like feature. As such, hole transfer from QDs to AZs tends to generate the spin-triplet-like charge

separated states, $[QD^{-}\text{-}AZ^{+}]^{3}$. The spin of $[QD^{-}\text{-}AZ^{+}]^{3}$ is then mostly preserved during CR2 to generate QD-^{3}AZ*.

Supplementary Note 2. Calculation of energy levels and charge transfer driving forces

AZ energy levels. The energy levels of AZs absorbed on CdS QDs were measured using cyclic voltammogram (CV) in our previous study.[1] From the measurement, the oxidation potential energy ($E_{AZ^{+}/AZ}$) is -4.3 eV (*vs.* vacuum), which corresponds to the HOMO level of AZ adsorbed on QD surfaces. In combination with the singlet and triplet transition energies of 2.16 (optical gap) and 1.27 eV, respectively, the estimated LUMO and triplet LUMO energy levels are at -2.14 and -3.03 eV (*vs.* vacuum). Note these are the electron energy levels in the presence of a hole in the HOMO, hence including the contribution of electron-hole binding energy in the molecule. In other words, the LUMO and triplet LUMO levels correspond to $E_{AZ^{+}/AZ*}$ and $E_{AZ^{+}/^{3}AZ*}$, respectively. The energy of direct electron injection into a neutral AZ molecule on QD surface should be even higher by hundreds of meV ($E_{AZ^{-}/AZ}$).

CdS QD energy levels. Ingole *et al.* measured the $1S_e$ energy level (E_e) of CdS QDs (with the 1*S* absorption peak at 410 nm) capped with oleic acid ligands using cyclic voltammetry (CV), and reported a value at -2.38 eV (*vs.* vacuum) for this sample.[11] On the basis of their result, we can estimate the $1S_e$ energy levels (E_e) in our QDs of various sizes according the following equations:[12]

$$E_e = E_{e,ref} + \frac{m_h}{m_e + m_h}\left[\left(E_g + E_{e-h}\right) - \left(E_{g,ref} + E_{e-h,ref}\right)\right] \qquad \text{(S1)},$$

$$E_{e-h} = -\frac{1.765e^2}{4\pi\varepsilon_0\varepsilon R} \qquad \text{(S2)},$$

where E_g is the optical band gap, $E_{e\text{-}h}$ is the electron-hole binding energy, *ref* indicates the values form the reference QDs, and m_e and m_h are the electron (0.2 m_0) and hole (0.7 m_0) effective masses, respectively. Using eqs. S1 and S2, E_e of our QDs are calculated to range from ~ -2.21 to -2.66 eV *vs.* vacuum, as Tabulated in Table S1.

CdSe NPL energy levels. Dey *et al.* measured CdSe NPLs (with the lowest absorption peak at 512 nm; 4 monolayer) capped with oleic acid ligands using X-ray photoelectron spectroscopy (XPS), and reported a valence band maximum at -5.66 eV (*vs.* vacuum) for this sample. The E_e of this sample can be calculated by adding the optical gap (2.42 eV) and electron-hole binding energy (0.33 eV)[13], resulting in E_e = -2.91 eV (*vs.* vacuum). Using this result as a reference, we can calculate E_e of NPLs of other thicknesses using eq. S1 (with m_e = 0.13 m_0 and m_h = 0.45 m_0). In these calculations, the values of $E_{e\text{-}h}$ in CdSe NPLs were adapted from the calculation by Efros et al., 413 meV, 330 meV, 278 meV and 242 meV for the 3, 4, 5, 6 monolayer NPLs, respectively.[13] With these parameters, E_e of our NPLs are calculated to range from ~ -2.66 to -3.20 eV *vs.* vacuum, as Tabulated in Table S1.

ET driving forces. The driving forces of electron transfer (ET) from photoexcited AZs to QDs (or NPLs) are calculated from the free energy changes between the states after and before the ET reaction. We follow the formalism in ref.[14] to calculate this free energy change. For the QD-AZ system, the initial and final states are AZ*-QD and AZ^+-QD^-, respectively. The free energy change is:

$$\Delta G_{ET} = E_{QD^-/QD} - E_{AZ^+/AZ} + E_{CS} \qquad \text{(S3)},$$

where E_{CS} is the electron-hole binding energy in the charge separated state (with a negative sign). Note the charging energies for QDs and AZs are already included into their redox

potential energies. For QDs, E_{CS} has been derived as:

$$E_{CS}(R) = -\int_0^R dr \int_0^\pi d\theta \int_0^{2\pi} d\varphi \frac{r^2 \sin\theta e^2 \rho(r)}{4\pi\varepsilon\sqrt{r^2\sin^2\theta + (R - r\cos\theta)^2}} \quad \text{(S4)},$$

where $\rho(r)$ is the charge density inside the QD and can be expressed as the following by assuming charge wavefunction for a particle in a sphere of infinite depth:

$$\rho(r) = e\left[\frac{\sin(\pi r/R)}{r\sqrt{2\pi R}}\right]^2 \quad \text{(S5)}.$$

A similar analytic expression for NPLs has not been derived due to the poorly-defined lateral sizes of the NPLs. In a related study, Efros et al. has calculated E_{CS} for CdSe nanorods (~-200 meV)[15], which have a similar dielectric-confinement-enhanced Coulombic effect as the NPLs studied here. Therefore, we adopted E_{CS} = -200 meV in our calculations.

Note that the ET driving force calculated from Eq. S3 is for the lowest energy conduction band level in the QDs or NPLs. However, there are many electronic levels in the conduction band; in addition to the lowest level, many other higher-lying level can possibly accept the electrons from photoexcited AZs as well. For example, for large-size QDs such as QD-450, ΔG_{ET} for the lowest level ($1S_e$) is -0.65 eV; considering that the $1P_e$ level is higher than the $1S_e$ level by ~0.27 eV (estimated from absorption spectrum), ΔG_{ET} for the $1P_e$ level is -0.38 eV. In this case, the observed ET rate should be the sum of all these allowed kinetic pathways. Due to this complication, a comparison of the size-dependent ET rates is not meaningful here.

CR driving forces. For the CR1 process in the CdS QD-AZ complexes, the initial and final states are AZ^+-QD^- and ground-state AZ-QD, respectively. The energy difference between these two states is related to ΔG_{ET} in Eq. S3 through:

$$\Delta G_{CR1} = -(E_{g,AZ*} + \Delta G_{ET}) \qquad \text{(S6)},$$

where $E_{g,AZ*}$ is the singlet transition energy for AZ molecules attached on QD surfaces (2.16 eV). The driving forces for CR1 in the CdSe NPL-AZ complexes can be calculated in the same way.

For the CR2 process in the CdS QD-AZ complexes, the initial and final states are AZ^{+}-QD^{-} and $^{3}AZ^{*}$-QD, respectively. The energy difference between these two states is related to ΔG_{CR1} in Eq. S6 through:

$$\Delta G_{CR2} = \Delta G_{CR1} + E_{g,{}^{3}AZ*} \qquad \text{(S7)},$$

where $E_{g,{}^{3}AZ*}$ is the triplet transition energy for AZ molecules attached on QD surfaces (1.27 eV).

The estimated E_e, ΔG_{ET}, ΔG_{CR1} and ΔG_{CR2} for CdS QDs of different sizes and CdSe NPLs of different thicknesses are summarized in Supplementary Tables 1.

Supplementary Table 1. Driving forces for QD-AZ and NPL-AZ complexes

		E_e (eV *vs* vac.)	ΔG_{ET} (eV)	ΔG_{CR1} (eV)	ΔG_{CR2} (eV)
CdS QDs	QD-391	-2.21	-0.27	-1.89	-0.62
	QD-400	-2.29	-0.34	-1.82	-0.55
	QD-412	-2.38	-0.42	-1.74	-0.47
	QD-423	-2.47	-0.49	-1.67	-0.40
	QD-431	-2.53	-0.54	-1.62	-0.35

	QD-441	-2.60	-0.60	-1.56	-0.29
	QD-450	-2.66	-0.65	-1.51	-0.24
CdSe NPLs	NPL-461	-2.66	-0.72	-1.44	NA
	NPL-510	-2.92	-0.98	-1.18	
	NPL-553	-3.10	-1.16	-0.99	
	NPL-578	-3.20	-1.26	-0.89	

Supplementary Note 3. Kinetics fitting models

For the experiments where AZs were selectively excited in the QD-AZ or NPL-AZ complexes, the kinetics of the exciton bleach (XB) feature of QDs or NPLs were fitted to the following multi-exponential equation:

$$S_{XB}(t) \propto A_{d,1}e^{-k_{d,1}t} + A_{d,2}e^{-k_{d,2}t} - A_{f,1}e^{-k_{f,1}t} - A_{f,2}e^{-k_{f,2}t} \quad \text{(S8)},$$

where $k_{d,i}$ ($A_{d,i}$) and $k_{f,i}$ ($A_{f,i}$) are the time constants (and the relative amplitudes of corresponding components) of the decay and formation processes, respectively, of the XB feature. The average rates were calculated by:

$$k_{ave} = (A_1k_1 + A_2k_2)/(A_1 + A_2) \quad \text{(S9)},$$

For the QD-AZ complexes selectively excited at the QDs, only the decay process due to CR2 was reveled at the XB feature:

$$S_{XB}(t) \propto A_{d,1}e^{-k_{d,1}t} + A_{d,2}e^{-k_{d,2}t} \quad \text{(S10)}.$$

The fitting parameters are tabulated in Supplementary Tables 2.

Supplementary Table 2. Multi-exponential fitting parameters for QD-AZ and NPL-AZ complexes

		Excite AZ						Excite QD
		$k_{f,1}$(ps^{-1}) ($A_{f,1}$)	$k_{f,2}$(ps^{-1}) ($A_{f,2}$)	$k_{d,1}$(ns^{-1}) ($A_{d,1}$)	$k_{d,2}$(ns^{-1}) ($A_{d,2}$)	k_{ET} (ps^{-1})	k_{CR1} (ns^{-1})	k_{CR2} (ps^{-1})
QD-AZ	QD-391	0.102 77.2%	0.0266 22.8%	2.99 89.0%	0.231 11.0%	0.0814 ±0.0025	2.08 ±0.05	0.228 ±0.006
	QD-400	0.0799 91.3%	0.0218 8.7%	2.32 80.4%	0.404 19.6%	0.0692 ±0.0122	1.50 ±0.14	0.196 ±0.007
	QD-412	0.179 38.8%	0.0308 61.2%	2.76 66.8%	0.650 33.2%	0.0845 ±0.075	1.42 ±0.30	0.142 ±0.004
	QD-423	0.230 30.4%	0.0348 69.6%	1.61 71.3%	0.339 28.7%	0.0901 ±0.0047	1.25 ±0.01	0.085 ±0.002
	QD-431	0.150 32.2%	0.0228 67.8%	1.17 67.2%	0.294 32.8%	0.0599 ±0.0075	0.882 ±0.017	0.054 ±0.002
	QD-441	0.139 34.0%	0.0207 66.0%	0.958 61.6%	0.244 38.4%	0.0570 ±0.006	0.684 ±0.002	0.030 ±0.006
	QD-450	0.079 44.2%	0.0131 55.8%	0.996 44.0%	0.219 56.0%	0.0383 ±0.0108	0.561 ±0.048	0.016 ±0.0005

NPL-AZ	NPL<br>-461	0.0801<br>51.5%	0.0166<br>48.5%	0.00101<br>84.3%	0.000093<br>15.7%	0.0488<br>±0.01002	0.0008<br>±0.00012	NA
	NPL<br>-510	0.0462<br>58.7%	0.0185<br>41.3%	0.00154<br>59.8%	0.00054<br>40.2%	0.0347<br>±0.00454	0.00114<br>±0.00052	
	NPL<br>-553	0.0803<br>60.7%	0.0171<br>39.3%	0.00218<br>12.1%	0.000724<br>87.9%	0.0552<br>±0.00163	0.0009<br>±0.00014	
	NPL<br>-578	0.1463<br>55.8%	0.0263<br>44.2%	0.000884<br>77.4%	0.00007<br>22.6%	0.0931<br>±0.0173	0.0007<br>±0.00006	

Supplementary Note 4. Calculation of molecular orbitals

The density functional theory (DFT) and time-dependent DFT (TDDFT) calculations in present study were carried out with Gaussian 16 software. The ground state geometries of free alizarin and model compound of CdS-surface-bound alizarin were optimized using hybrid functional B3LYP, and then the frequency calculations at the same levels of theory were performed to confirm that each optimized structure was the real minimum. The vertically excited energies (VEEs) were computed using linear-response TD-DFT method based on the optimized ground state geometries. The TZVP basis set was adopted and the solvation effects were treated by the integral equation formalism version of the polarizable continuum model. The D3 version of Grimme's dispersion[16] was included to account for the dispersion forces.

The accuracy of the calculation was established by comparing the computed transition energies to the experimental ones, as detailed in our previous study[1]. Specifically, the

computed transition energy of the S_1 state of free AZs in toluene is 2.84 eV, agreeing well with the experimental result (2.82 eV). The computed transition energy of the S_1 state of model compound of CdS-surface-bound AZs is 2.16 eV, which is also in reasonable agreement with the experimental result (2.05 eV).

Supplementary Fig. 7a shows the optimized geometry of the model compound of CdS-surface-bound AZ. The two Cd atoms chelated by the AZ molecule are indicated. By setting the line along the two Cd atoms as the X-axis, the Y-axis is the normal of the QD-AZ interface along which electronic coupling takes place. Based on the DFT computed wavefunctions of S_0 and T_1 states, their corresponding electron densities were first computed and then integrated to reveal the electron density distributions along the Y-axis by employing the MultiWfn software[17] using the following equation:

$$I_L(y) = \int_{-\infty}^{+\infty}\int_{-\infty}^{+\infty} p(x,y,z)dxdz \qquad \text{(S11).}$$

where $p(x,y,z)$ was the computed electron density. The results for S_0 and T_1 states are plotted in Supplementary Fig. 7b for comparison, revealing almost the same electron density distributions for these two states. The same conclusion was found along other axes.

Supplementary Note 5. Calculation of electron density at the NC surface

As we discussed in the main text, the electronic coupling term ($|V|^2$) for QDs of various sizes is not a constant but rather is proportional to the electron probability density at the NC surface which can be expressed as the square of wavefunction at the NC surface ($|\Psi_s|^2$). In order to calculate $|\Psi_s|^2$, we applied an effective mass approximation (EMA) model and treated the electron in QDs and NPLs as a particle confined in spherical and rectangular wells,

respectively, of finite depth. The confinement depth for the electron in QDs and NPLs is determined by the conduction band edge of the semiconductor and the lowest unoccupied molecular orbital (LUMO) of ligand molecules (~ -1 eV *vs.* vacuum for oleic acid). Full parameters for the calculation were reported in ref[18]. In order to validate the accuracy of the simple model, we examined the size-dependent optical gaps of CdS QDs calculated from the first quantized electron and hole levels and accounting for the electron-hole binding as a perturbation, which agrees reasonably well with our experimental data (Supplementary Fig. 8a). Thus, this simple EMA model is likely sufficient to describe the energies and wavefunctions of the first quantized electron and hole states. Moreover, because $|\Psi_s|^2$ depends upon the tunneling barrier (i.e., the offset between CdS CB and ligand LUMO), we examined the impact of the uncertainty of the barrier height on $|\Psi_s|^2$. As shown in Supplementary Fig. 8b, changing the OA LUMO from -0.75 to -1.25 eV (*vs.* vacuum) does not lead to noticeable changes to the size-dependent amplitudes of $|\Psi_s|^2$. The reason is that the barrier is already high (~2.84 eV in the case of OA LUMO at -1 eV), and hence a variation of 0.5 eV does not strongly perturb the tunneling probability.

Note that in the case of NPLs, because of the much larger area of the basal planes compared with the lateral facets, we assume that the AZ molecules are mostly adsorbed on the basal planes. Therefore, the $|\Psi_s|^2$ for NPLs is obtained by integrating the calculated wavefunction squares over the basal plane areas and thus it has a unit of nm^{-1} instead of nm^{-3} for the QD case.

The calculated size-dependent $|\Psi_s|^2$ values were used to scale the measured CR rates (k_{CR}) such as to obtain coupling-strength-scaled CR rates ($k_{CR,s}$) via: $k_{CR,s} = k_{CR}/|\Psi_s|^2$; See

Supplementary Tables 3.

Supplementary Table 3. Size-dependent surface electron density and scaled CR rates

		$\|\Psi_s\|^2$ (nm^{-3})	k_{CR1} (ps^{-1})	k_{CR2} (ps^{-1})	Scaled k_{CR1} ($ps^{-1}nm^3$)	Scaled k_{CR2} ($ps^{-1}nm^3$)
QD-AZ	QD-391	0.060	0.0021	0.228	0.0347	3.80
	QD-400	0.045	0.0015	0.196	0.0332	4.36
	QD-412	0.032	0.0014	0.142	0.0445	4.44
	QD-423	0.022	0.0012	0.085	0.0567	3.86
	QD-431	0.017	0.0009	0.054	0.0519	3.18
	QD-441	0.011	0.0007	0.030	0.0622	2.73
	QD-450	0.008	0.0006	0.016	0.0721	2.16
		$\|\Psi_s\|^2$ (nm^{-1})	k_{CR1} (ps^{-1})	k_{CR2} (ps^{-1})	Scaled k_{CR1} ($ps^{-1}nm$)	Scaled k_{CR2} ($ps^{-1}nm$)
NPL-AZ	NPL-461	0.393	0.0008	NA	0.00211	NA
	NPL-510	0.244	0.00114		0.00469	
	NPL-553	0.156	0.0009		0.00558	
	NPL-578	0.104	0.0007		0.00704	

References for SI:

1 Wang, J., Ding, T., Nie, C., Wang, M., Zhou, P. & Wu, K. Spin-Controlled Charge Recombination Pathways across the Inorganic/Organic Interface. *J. Am. Chem. Soc.* **142**, 4723-4731 (2020).

2 Kim, J., Wong, C. Y. & Scholes, G. D. Exciton Fine Structure and Spin Relaxation in Semiconductor Colloidal Quantum Dots. *Acc. Chem. Res.* **42**, 1037-1046 (2009).

3 Wong, C. Y., Kim, J., Nair, P. S., Nagy, M. C. & Scholes, G. D. Relaxation in the Exciton Fine Structure of Semiconductor Nanocrystals. *J. Phys. Chem. C* **113**, 795-811 (2009).

4 Tong, H., Feng, D., Li, X., Deng, L., Leng, Y., Jia, T. & Sun, Z. Room-Temperature Electron Spin Generation by Femtosecond Laser Pulses in Colloidal CdS Quantum Dots. *Materials* **6** (2013).

5 Li, X., Feng, D., Tong, H., Jia, T., Deng, L., Sun, Z. & Xu, Z. Hole Surface Trapping Dynamics Directly Monitored by Electron Spin Manipulation in CdS Nanocrystals. *J. Phys. Chem. Lett.* **5**, 4310-4316 (2014).

6 Hu, R., Yakovlev, D. R., Liang, P., Qiang, G., Chen, C., Jia, T., Sun, Z., Bayer, M. & Feng, D. Origin of Two Larmor Frequencies in the Coherent Spin Dynamics of Colloidal CdSe Quantum Dots Revealed by Controlled Charging. *J. Phys. Chem. Lett.* **10**, 3681-3687 (2019).

7 Efros, A. L., Rosen, M., Kuno, M., Nirmal, M., Norris, D. J. & Bawendi, M. Band-edge exciton in quantum dots of semiconductors with a degenerate valence band: Dark and bright exciton states. *Phys. Rev. B* **54**, 4843-4856 (1996).

8 Norris, D. J., Efros, A. L., Rosen, M. & Bawendi, M. G. Size Dependence of Exciton Fine Structure in CdSe Quantum Dots. *Phys. Rev. B* **53**, 16347-16354 (1996).

9 Ghosh, T., Dehnel, J., Fabian, M., Lifshitz, E., Baer, R. & Ruhman, S. Spin Blockades to Relaxation of Hot Multiexcitons in Nanocrystals. *J. Phys. Chem. Lett.* **10**, 2341-2348 (2019).

10 Gündoğdu, K., Hall, K. C., Koerperick, E. J., Pryor, C. E., Flatté, M. E., Boggess, T. F., Shchekin, O. B. & Deppe, D. G. Electron and hole spin dynamics in semiconductor quantum dots. *Appl. Phys. Lett.* **86**, 113111 (2005).

11 Ingole, P. P., Markad, G. B., Saraf, D., Tatikondewar, L., Nene, O., Kshirsagar, A. & Haram, S. K. Band Gap Bowing at Nanoscale: Investigation of CdSxSe1–x Alloy Quantum Dots through Cyclic Voltammetry and Density Functional Theory. *J. Phys. Chem. C* **117**, 7376-7383 (2013).

12 Brus, L. E. A simple model for the ionization potential, electron affinity, and aqueous redox potentials of small semiconductor crystallites. *J. Chem. Phys.* **79**, 5566-5571 (1983).

13 Benchamekh, R., Gippius, N. A., Even, J., Nestoklon, M. O., Jancu, J. M., Ithurria, S., Dubertret, B., Efros, A. L. & Voisin, P. Tight-binding calculations of image-charge effects in colloidal nanoscale platelets of CdSe. *Phys. Rev. B* **89**, 035307 (2014).

14 Lai, R., Liu, Y., Luo, X., Chen, L., Han, Y., Lv, M., Liang, G., Chen, J., Zhang, C., Di, D., Scholes, G. D., Castellano, F. N. & Wu, K. Shallow distance-dependent triplet energy migration mediated by endothermic charge-transfer. *Nat. Commun.* **12**, 1532

(2021).
15 Yang, Y., Wu, K., Shabaev, A., Efros, A. L., Lian, T. & Beard, M. C. Direct Observation of Photoexcited Hole Localization in CdSe Nanorods. *ACS Energy Letters* **1**, 76-81 (2016).
16 Grimme, S., Antony, J., Ehrlich, S. & Krieg, H. A consistent and accurate ab initio parametrization of density functional dispersion correction (DFT-D) for the 94 elements H-Pu. *J. Chem. Phys.* **132**, 154104 (2010).
17 Lu, T. & Chen, F. Multiwfn: A multifunctional wavefunction analyzer. *Journal of Computational Chemistry* **33**, 580-592 (2012).
18 Zhu, H., Yang, Y., Hyeon-Deuk, K., Califano, M., Song, N., Wang, Y., Zhang, W., Prezhdo, O. V. & Lian, T. Auger-Assisted Electron Transfer from Photoexcited Semiconductor Quantum Dots. *Nano Lett.* **14**, 1263-1269 (2014).
